# Breaking the cycle: a comprehensive exploration of topical steroid addiction and withdrawal

**DOI:** 10.3389/falgy.2025.1547923

**Published:** 2025-03-31

**Authors:** Anish R. Maskey, Akimi Sasaki, Manuel Sargen, Maureen Kennedy, Raj K. Tiwari, Jan Geliebter, Bijan Safai, Xiu-Min Li

**Affiliations:** ^1^ Department of Pathology, Microbiology, & Immunology, New York Medical College, Valhalla, NY, United States; ^2^ Department of Public Health, School of Health Sciences & Practice, New York Medical College, Valhalla, NY, United States; ^3^ Department of Dermatology, New York Medical College, Valhalla, NY, United States; ^4^ Department of Otolaryngology, New York Medical College, Valhalla, NY, United States

**Keywords:** topical steroid withdrawal, topical steroid addiction, corticosteroids, atopic dermatitis, treatment and management

## Abstract

Topical steroid withdrawal (TSW) is a skin condition characterized by red burning, itchy, painful skin lesions, often accompanied by peeling, and cracking. Patients experience sleep disturbances due to intense itching, significantly impacting their quality of life. A majority of affected individuals develop secondary bacterial infection, marked by heavy colonization of *Staphylococcus aureus (S. aureus)* and alterations in the skin microbiome. TSW is described as a rebound effect following discontinuation of prolonged use of mid-to-high-potency topical corticosteroids. There exist no definitive diagnostic criteria for this entity, and it is often misdiagnosed as a flare-up of an underlying condition or a contact allergy. Despite numerous personal reports and experiences shared on online platforms, studies on TSW remain scarce in scientific literature. Recognizing and effectively managing this condition is critical for healthcare providers seeking to develop comprehensive management plans. These plans typically include supportive therapy for both physical and psychological symptoms, as well as the gradual tapering of corticosteroid use before complete discontinuation. This review aims to consolidate the existing knowledge on TSW, providing a comprehensive resource for its identification, management, and treatment. By enhancing understanding of TSW, this review seeks to support healthcare providers in implementing optimal management strategies and improving patient outcomes.

## 1 Introduction

Atopic dermatitis (AD), a prevalent inflammatory skin disorder, affects an estimated 17% of children and 7% of adults in the United States (1). Redness, swelling, cracking, crusting, scaling due to intense itching and pruritus are key hallmarks of AD, Topical corticosteroids (TCS, or TS) have been the mainstays in managing AD for more than 40 years and are endorsed as the first-line anti-inflammatory treatment for eczema and other atopic conditions in international guidelines (1, 2). Despite their demonstrated safety and efficacy in both short-term daily use and long-term intermittent application, concerns have surfaced regarding the cumulative effects of prolonged TCS use, specifically regarding topical steroid addiction (TSA) and withdrawal (TSW) (3), with numerous websites and patient blogs warning against these potential risks (4).

TSA and TSW are adverse outcomes associated with inappropriate or prolonged TCS use. Even though TCS has been used for AD for more than 40-years, and despite the term “steroid addiction” being introduced in 1979 (5), TSA and TSW have only recently gained attention on online platforms. TSA tends to precede TSW (6) and is defined as physical dependence on TCS with prolonged use (5). Patients with TSA typically present with increasing resistance and may require more potent steroids or frequent applications to prevent flares (7, 8). Furthermore, TSA is also characterized by the exacerbation of dermatological conditions following TCS withdrawal compared to pre- application (9, 10). This cycle of increasing dependency on TSA is often referred to as “steroid addiction syndrome” (4, 8). On the other hand, TSW, topical steroid withdrawal syndrome (TSWS), or “red skin syndromes” specifically refers to manifestations occurring after TCS cessation (2, 11) and usually results from long-term application of moderate-to-high potency TCS on sensitive areas such as the face, genitals, and intertriginous regions. In severe cases, the skin lesion extends to the entire body. It is often depicted as severe cutaneous rebound inflammation and new distressing symptoms upon TCS discontinuation (3, 12, 13). Various terms have been used to describe TSA/TSW, including, but not limited to, “red skin syndromes” (2, 11), “red face” (4), and “red scrotum” (4). The face is the most affected area in TSA/TSW; however, other areas with high percutaneous penetration, such as the genitals and intertriginous regions, are more likely to be affected as well (3, 4, 10, 12).

**Figure 1:**
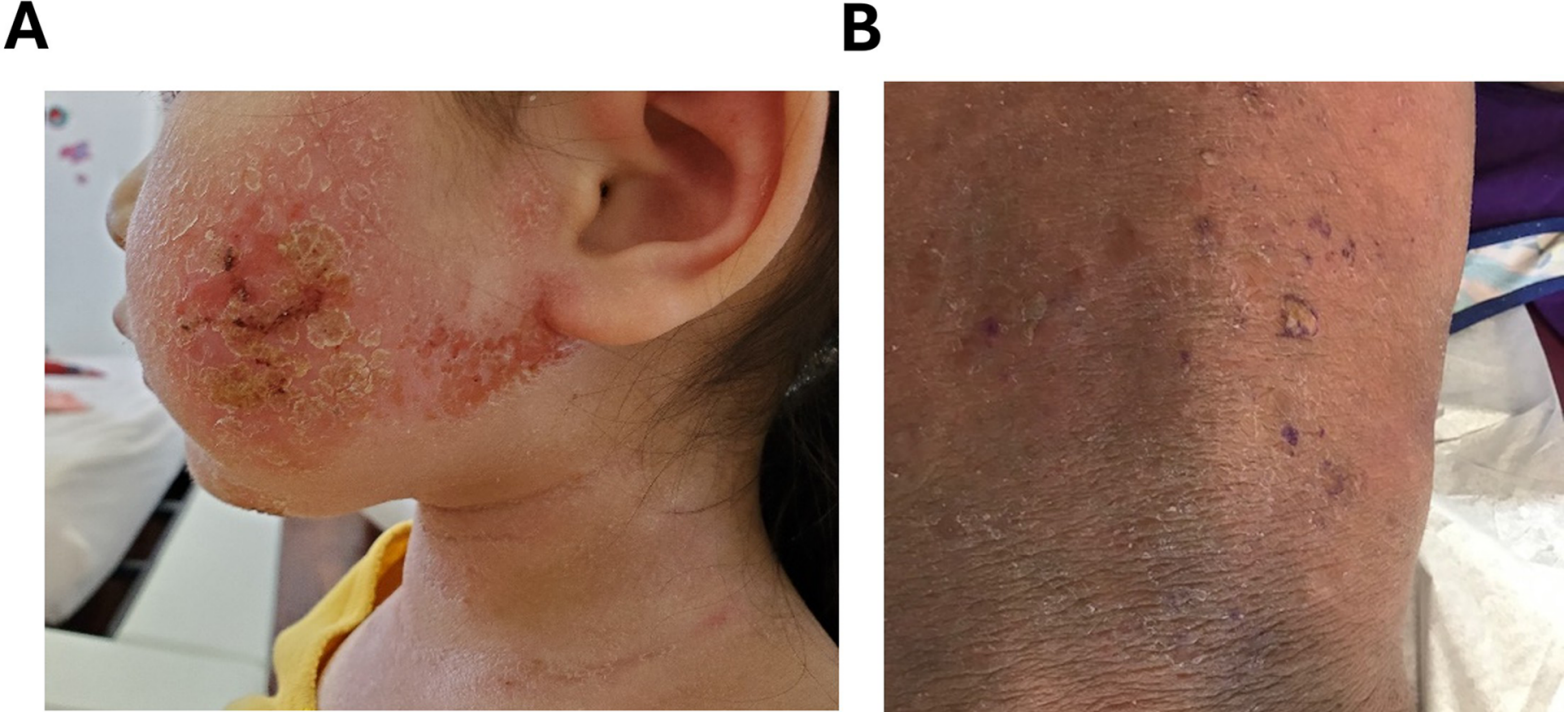
**(A)** Erythematoedematous subtype of steroid withdrawal characterized by severe oozing, crust, redness and excoriation. **(B)** Papulopustular subtype of steroid withdrawal with erythema, papules and pustules. *Permission obtained to reuse figure from* (14).

Two primary morphological subtypes of TSW have been identified: erythematoedematous (47.9%) (Figure 1A) and papulopustular (52.1%) (Figure 1B) (4). The erythematoedematous subtype is prevalent among patients with chronic eczematous conditions such as AD and seborrheic dermatitis. It is characterized by erythema, scaling, edema, and burning sensations. Conversely, the papulopustular subtype is more common in patients using TCS for the treatment of cosmetic, pigmentary, or acneiform conditions and frequently occurs in those who develop steroid rosacea, although its presence is not required for diagnosis. As the name suggests, the papulopustular subtype typically presents with erythema, papules, and pustules and is associated with less frequent occurrences of swelling, edema, burning and stinging (4). While clinical overlaps between the two variants exist, the erythematoedematous subtype is distinguished by more pronounced burning, stinging sensations, and edema. Specifically, burning sensations and edema are present in 94.6% and 43.3% of erythematoedematous cases, respectively, compared to 35.4% and 2.9% of papulopustular cases (4). The terms “topical steroid induced facial rosaceiform dermatitis” (15) and “topical corticosteroid-induced rosacea-like dermatitis” (16) have been used to describe patients experiencing erythema and telangiectasias with prolonged TCS use. These conditions are believed to correspond with the erythematoedematous subtype of TSW (13).

Despite TSA/TSW being recognized in 1979, and patients reporting it on online platforms, research on these conditions has been limited. Furthermore, an absence of consensus in diagnostic criteria contributes to the incomplete understanding of these conditions within the dermatological community (2, 3). Therefore, a more comprehensive understanding of TSA/TSW among medical professionals is required to mitigate the burden of these conditions. Accordingly, this review summarizes key information regarding TSA/TSW.

## 2 Epidemiology and risk factors

Determining the prevalence of TSA/TSW is challenging due to the lack of clear diagnostic criteria and significant variation in literature reports. For example, a Japanese study conducted in 2000 found that 12% of patients with AD using TCS were experiencing TSA (9). On the other hand, a multinational study involving 2,160 subjects with eczema (aged 18 years or older or caregivers of children with eczema) reported that 79% of adults and 43% of caregivers observed symptoms consistent with TSW in themselves and in their children, respectively (3).

While determining the prevalence of TSA/TSW is challenging, more is known about the associated risk factors. Increased TCS potency has been identified as a risk factor for TSA/TSW, with moderate- to high-potency TCS use being associated with an increased likelihood of disease (2–4, 13). Mid- or high-potency TCS use has been reported in 98.6% of patients with TSW (4), and in 73% of children with AD demonstrating symptoms of TSW (12). The vasoconstrictor assay categorizes TCS into seven potency groups, ranging from ultra-high potency (Group I) to low potency (Group VII) based on the extent of cutaneous vasoconstriction (17). For example, mometasone is a medium potency steroid (class IV–V) with strong topical efficacy but limited systemic absorption, making it more suitable for long term use. In contrast, clobetasol propionate is ultra-high potency (class I) with significantly higher penetration and ability to cause irritation, skin atrophy, striae and systemic side effects such as hypothalamic-pituitary-adrenal suppression, glaucoma, septic necrosis of the femoral head, hyperglycemia, hypertension (17). Given that a high proportion of patients with TSA/TSW have used mid- or high- potency TCS and more potent molecules are associated with greater dependence (18), caution must be practiced when prescribing these medications. Being mindful of TCS potency is particularly important in pediatric populations, who have a larger body surface area (BSA)-to-weight-ratio, promoting greater TCS absorption and increased systemic effects such as hypothalamic-pituitary-adrenal (HPA) axis suppression. Mid-potency TCS may be a safer alternative to high-potency TCS when treating TSW in these populations, as they do not affect the HPA axis when used “up to 3 times a day for 4 weeks or twice daily for 16 weeks” (19). Furthermore, the use of a topical steroid ladder, which classifies TCS based on potency, may be a useful tool in facilitating tapering strategies and minimizing adverse effects in both adult and pediatric patient population (20).

Similarly, dosage also influences the risk of TSW, with the use of multiple TCSs increasing the likelihood of its occurrence. A previous study reported that, among 1,702 participants with symptoms consistent with TSW, 82% experienced TSW symptoms when using two TCSs, compared to 64% with one TCS (3). Not only does TCS dosage matter, but total corticosteroid use also plays a role. History of oral corticosteroid use may indicate more severe clinical conditions and is associated with TCS overuse, contributing to a greater likelihood of TSW (21). Lastly, longer duration of TCS application is another critical factor; 86% of individuals using TCS for 6 or more years reported symptoms consistent with TSW, compared to 53% of individuals using TCS for less than 1 year (3). Generally, the duration of TCS usage is 6 months or more (13), with 85.2% of patients with TSW reporting use longer than 12 months (4).

Certain patient factors, such as gender, primary concern, and region of application, have also been identified to increase risk. Individuals with AD are particularly susceptible to TSW, with AD being the initial indication for TCS use in one-third of cases. Furthermore, adult women are the largest demographic, comprising 81% of cases, and nearly all patients with TSW (97%) having a history of applying TCS to the face (4). The reason for the predominance in women is not fully understood (13), but it is believed to result from TCS use related to cosmetic and pigmentary concerns of the face (22). In particular, the anti-melanogenic properties of TCS may be favorable in those seeking skin lightening, contributing to misuse and subsequent TSA/TSW (13, 22). Additionally, patient age, accessibility of TCS, and behavior can also be risk factors. Studies show that younger patients are associated with an increased sensitivity to TCS and are likely to develop TSA/TSW. Unlike adults, who typically develop TSW after 6 months of TCS use (13), children can develop TSW after just 2 months of use, with symptoms potentially persisting for over 12 months (12). Studies show that recovery rates and prognosis also differ between children and adults, with 44% of children recovering from TSW compared to 28% of adults, and 8% of adults experiencing symptoms for more than 5 years (3). Additionally, TCS misuse is more prevalent in developing countries due to unregulated and over-the-counter sales of TCS and self-treatment, often for cosmetic purposes or non-responsive dermatoses. Moreover, patient behaviors such as self-treatment, seeking prescriptions from practitioners other than dermatologists, reusing old prescriptions for recurrent or similar rashes, and sharing prescriptions with others are concerning and can increase one's susceptibility (22). Furthermore, clinical evidence suggests that young females, especially those with skin of color are predominantly affected by this condition owing their vulnerability to TCS misuse for cosmetic reasons, such as use of fairness creams (22).

Lastly, an often-overlooked risk factor for TSW is occupational exposure to steroids. Specifically, healthcare professionals treating eczema patients may be exposed to TCS when applying them to patients, which can lead to skin irritation or allergic reactions if proper handling procedures are not followed—particularly with repeated exposure to high-potency steroids. This exposure can be mitigated if appropriate safety measures are taken. Wearing protective gloves, maintaining proper hand hygiene, minimizing direct skin contact, and employing correct application techniques are essential strategies to reduce the likelihood of developing TSW due to occupational exposure. Furthermore, occupational contact allergy, often presented as chronic irritative hand dermatitis, is primarily caused by water exposure or humidity in gloves, and corticosteroid use in such cases may be problematic. In these cases, protection and allergen avoidance are of utmost importance and corticosteroids should be the last choice. Alternative treatments such as tacrolimus and delgocitinib could be considered for its management.

## 3 Pathophysiology

**Figure 2:**
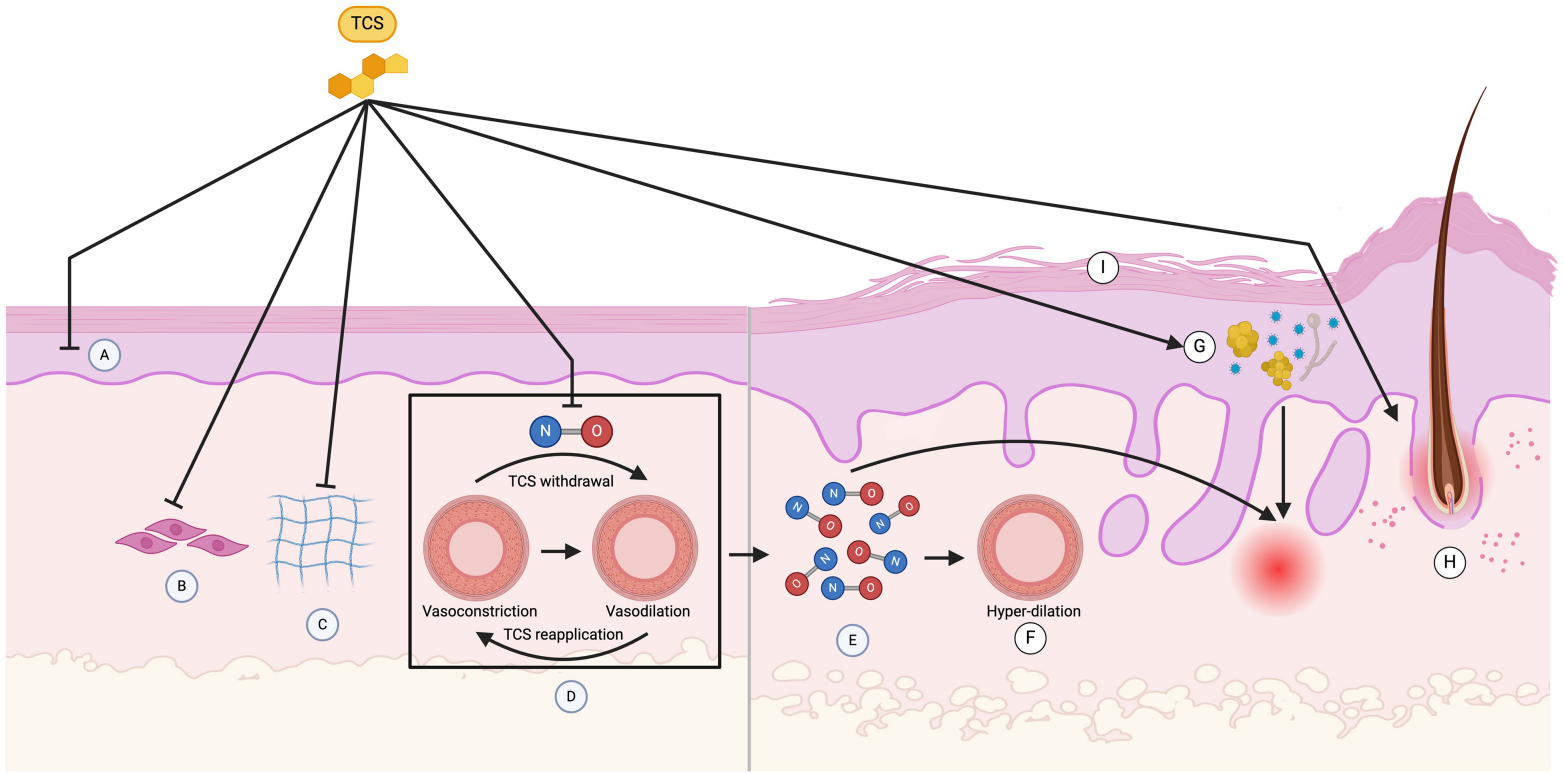
Pathophysiology of topical steroid addiction (TSA) and withdrawal (TSW). Exogenous application of topical corticosteroids (TCS) suppresses intrinsic cortisol production by keratinocytes **(A)** through negative feedback mechanisms involving the downregulation of enzymes like CYP11A1, CYP17A1, 3βHSD1, CYP21, and CYP11B1. Upon TCS withdrawal, the elimination of exogenous cortisol and reduced enzymatic activity leads to a temporary reduction in cortisol production, worsening inflammation in TSW. TCS also inhibits the proliferation and extracellular matrix protein synthesis of keratinocytes and fibroblasts **(B)**, reducing steroidogenesis of epidermal lipids such as ceramides, cholesterol, and fatty acids, resulting in a thinned stratum corneum and increased transepidermal water loss (I). Skin atrophy arises from TCS-induced reduction in collagen synthesis **(C)**, caused by decreased prolyl hydroxylase activity and collagen mRNA degradation. Chronic TCS use also inhibits endothelial nitric oxide (NO) production, leading to vasoconstriction, which reverses upon TCS withdrawal, causing excess NO release and subsequent vasodilation **(D)**. The vasodilation is associated with adverse effects such as erythema, itching and burning. Reapplication of TCS is often sought to reverse these adverse effects, resulting in a cycle of vasoconstriction and vasodilation **(D)**, termed the “trampoline effect” or “neon sign,” and excess NO accumulation **(E)**, leading to hyperdilation of blood vessels beyond their pre-steroid state **(F)**. This excess NO and prolonged hyperdilation are thought to contribute significantly to the erythema characteristic of TSA/TSW. Prolonged TCS use induces immunosuppression as well, making the skin more susceptible to the overgrowth of microorganisms. When TCS are withdrawn, these microorganisms can act as superantigens, inducing cytokine release and subsequent inflammation **(G)**. Papulopustular and acneiform lesions, characteristic of the papulopustular subtype of TSW, are thought to result from TCS degradation of the follicular epithelium and subsequent extrusion of follicular contents **(H)**. These interconnected processes illustrate the impact of TCS on keratinocyte and fibroblast function, endothelial response, immune function, and follicular epithelium and skin barrier integrity, ultimately driving the pathological changes observed in TSA and TSW (Created with BioRender).

The pathophysiology of TSA/TSW is not fully understood. However, previous research and literature suggest that the progression of TSA/TSW is multifactorial. Some of the key contributing factors are outlined in Figure 2.

### 3.1 Dysregulation of endogenous cortisol production by keratinocytes

Former studies have revealed that keratinocytes possess all the necessary mRNA and enzymes for the metabolic conversion of cholesterol to cortisol, suggesting the presence of a non-adrenal steroidal system in human skin (23, 24). During TSA, exogenous application of TCS may thus suppress the intrinsic production of cortisol by keratinocytes (Figure 2A) through negative feedback mechanisms. This could involve the downregulation of enzymes involved in cortisol synthesis in keratinocytes such as CYP11A1, CYP17A1, 3bHSD1, CYP21, and CYP11B1 (24). It is likely that TCS withdrawal results in elimination of exogenous cortisol, and the suppressed enzymatic activity of keratinocytes may take a while to return to baseline levels, resulting in a period of decreased cortisol production. Given that cortisol produced from keratinocytes helps to modulate inflammatory reactions, the temporary reduction in cortisol may be responsible for the worsening inflammation seen in TSW.

### 3.2 Skin atrophy

TCS suppress proliferation and the extracellular matrix (ECM) protein synthesis of keratinocytes (Figure 2A) and fibroblasts (Figure 2B), which can be viewed histopathologically. This inhibition results in reduced steroidogenesis of epidermal lipids such as ceramides, cholesterol, and fatty acids. As a result, the stratum corneum is thinned (Figure 2I), and greater transepidermal water loss (TEWL) transpires. The resultant skin has reduced skin barrier function, tensile strength, and elasticity (25), which could correspond to the formation of “elephant wrinkles” seen in TSA/TSW.

Similarly, skin atrophy may also occur from reduced collagen synthesis (Figure 2C). TCS indirectly inhibits collagen synthesis by reducing prolyl hydroxylase activity or promoting collagen mRNA degradation (25). They also directly inhibit synthesis by reducing procollagen gene expression through glucocorticoid response elements that negatively regulate Smad proteins, which are necessary for type I collagen transcription (25). Additionally, they may promote epidermal atrophy by overexpression of tissue proteases and keratinocyte proteins (12). For instance, TCS increases thymic stromal lymphopoietin activity, which shifts the T-helper (Th) lymphocyte ratio from a balanced Th1/Th2 population to Th2 predominance, like that seen in AD (12).

### 3.3 Role of nitric oxide

Nitric oxide (NO), an endothelium-derived relaxing factor (EDRF), released by vascular endothelial cells, functions as a potent vasodilator (26). Chronic TCS use inhibits endothelial NO, causing persistent vasoconstriction. Following TCS removal, accumulated endothelial NO is released, resulting in vasodilation and TSW symptoms such as erythema, itching, and burning sensation. Reapplying TCS to alleviate these unwanted symptoms results in vasoconstriction. The dependency on TCS results in alternating cycles of vasoconstriction and vasodilation, known as the “trampoline effect” or “neon sign,” (Figure 2D) and leads to excess NO accumulation (Figure 2E) (22) and hyperdilation of blood vessels beyond their pre- steroid diameter (Figure 2F) (22, 27). In summary, excess NO results in prolonged hyperdilation of vessels in TSA/TSW and is thought to contribute to the cardinal sign of erythema.

While NO is believed to contribute to the pathophysiology of TSA/TSW, its effects are difficult to discern and are inconclusive. Previous studies have demonstrated higher serum NO levels in inflammatory skin diseases like AD (10, 28), making it challenging to identify if elevated NO is due to TSA/TSW or the underlying condition. Furthermore, NO acts as a signaling molecule with both pro-inflammatory and anti-inflammatory effects depending on the context of its production. It is anti-inflammatory under normal physiological conditions (29) and participates in various biological functions including barrier homeostasis, wound healing, and antimicrobial defense (30). On the other hand, it is also pro- inflammatory and can participate in cutaneous inflammation in pathological conditions (29, 30). Topical NO-releasing products have also been shown to improve AD symptoms in humans and murine models (30). The dual nature of NO's effects in inflammation complicates the understanding of its role in TSA/TSW. However, despite its contradictory effects, NO levels have been found to be elevated in patients with TSA compared to cured patients and patients with eczema, indicating that serum NO may be useful in identifying addicted individuals (31). Enhancing our understanding of NO and its role in the pathology of TSA/TSW, as well as other inflammatory conditions like atopic dermatitis, could lead to the development of more effective treatments.

### 3.4 Topical corticosteroid-induced immunosuppression

Prolonged TCS use induces immunosuppression, making the skin more susceptible to the overgrowth of microorganisms. This may initially be helpful to patients with AD and other skin conditions, as a skin microbiome lacking biodiversity has been linked to many skin problems (32). However, when TCS are discontinued or withdrawn, these flourishing microorganisms can act as superantigens. As a result, the immune system which is no longer being suppressed induces cytokine release in response to the microbes and subsequent inflammation (Figure 2G) (18, 22, 32).

### 3.5 Direct effects on follicular epithelium

Papulopustular and acneiform lesions, characteristic of the papulopustular subtype, are thought to result from TCS degradation of the follicular epithelium and subsequent extrusion of follicular contents (Figure 2H) (22).

### 3.6 Glucocorticoid receptor expression

TCS acts by binding to intracellular glucocorticoid receptors (GR), of which two splicing variants have been identified, the GRa and GRb isoforms (10). Patients with a poor response following TCS treatment were shown to have upregulated GRb expression, while patients with a good response showed no changes in expression (10). Having higher GRb expression is thought to correlate to TCS insensitivity (10). Patients exhibiting TCS insensitivity may consequently use higher doses of TCS for extended periods, thereby elevating their risk of developing TSA/TSW.

### 3.7 Cytokines

TCS withdrawal has been associated with upregulation of IL1-α TNF-α, inhibitor of nuclear factor kappa-B kinase subunits alpha and beta (IKK1, IKK2) and nuclear factor kappa-B (NF-kB) in the epidermis. These cytokines gradually diminish after 1 week (33).

## 4 Clinical features of TSA/TSW

### 4.1 Signs and symptoms

**Figure 3:**
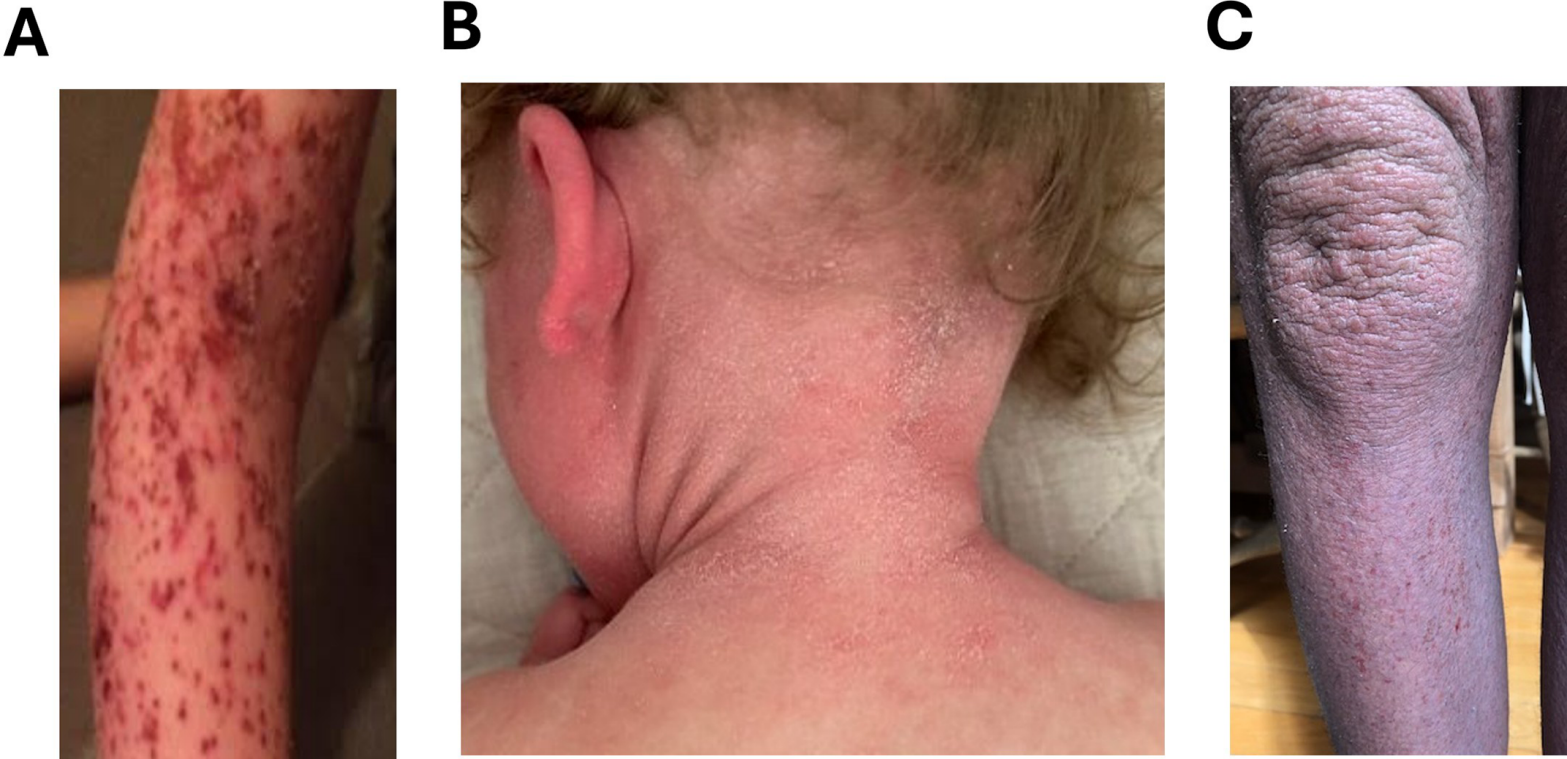
Signs and symptoms of TSW. **(A)** Widespread redness in the arm. **(B)** Serious redness in the ear. **(C)** Elephant wrinkles. *Permission obtained to reuse figure from* (14).

When patients with TSA discontinue TCS, they may experience widespread erythema (Figure 3A), the most common sign of TSW. The erythema can extend beyond original eczematous areas to previously untreated sites (22) and be accompanied by other signs associated with TSW such as the headlight sign (Figure 3B), red sleeve sign, and “elephant wrinkles” (Figure 3C). Patients undergoing TSW commonly exhibit symptoms such as intense burning sensations, pruritus, and edema, amongst others (4). Clinical signs and symptoms of TSW described in literature are summarized in Table 1.

**Table 1:** Clinical signs and symptoms.

Major dermatological signs	Features
Erythema (cardinal sign) (3, 4, 9, 11, 13)	• May appear less pronounced (22) or occur as hyperpigmentation depending on skin tone (3) • May extend beyond the initial application site or spread to distal sites (22) • Reported in 92.3% of patients with TSW (4)
Headlight sign (2, 4, 13)	• Distinct demarcation between erythematous and normal skin, in which redness normally ends at mid-cheek • The nasal, perinasal, and perioral areas are spared
Red sleeve (3)	• Rebound eruption of the extremities with a sharp cutoff at the margin of the dorsal and palmer (or solar) sides (21) • May appear dark depending on skin tone (3)
“Elephant wrinkles” (2, 3, 13)	• Thickened skin with diminished elasticity, commonly affecting extensor surfaces such as the anterior knees, elbows and neck (2, 13)
Other dermatological signs and symptoms	Features
Burning or stinging skin (3, 4, 9, 11)	• Affects 94.6% of the erythematoedematous type (4)
Skin peeling, flaking, or exfoliation (3, 4, 9, 11)	• Reported in 33.3% of patients with TSW (4)
Pruritis (3, 4, 9, 11)	• Reported in 45.4% of patients with TSW (4)
New skin hypersensitivity or dysesthesias (to various materials or environmental conditions) (3, 4, 13)	• Sensitivity to water, movement, moisturizers, fabric, temperature, sunlight (3) • Facial hot flashes are a common symptom of TSW (4) • Dysesthesia reported in 13.4% of patients with TSW (13)
Papulopustules (4)	• Papules ± nodules reported in 61.6% of patients with TSW (4) • More prevalent in the papulopustular type (4) • Often accompany vascular changes due to TCS use, attributed to focal degeneration and inflammatory reactions in the intrafollicular and perifollicular areas (22)
Hyperhidrosis or itchy wheals	• Signify recovery (9)
Alopecia (3)	• Can involve the head and/or body
Skin oozing or weeping (3, 4, 9, 11)	• Less common, reported in 1% of patients with TSW (4)
Changes to skin of the genitalia (11)	• Burning erythema ± pain of the scrotum, inguinal and penile area that may be accompanied by atrophy of the glans with disease progression • May be described in males as “red scrotum syndrome” • Pruritis vulvae and vulvodynia in females
Skin atrophy (2, 22)	• Increased skin transparency and sheen (22)
Telangiectasia (2, 3, 8, 13, 22)	• Dermal atrophy allows easier visualization of dermal capillaries
Non-dermatological signs and symptoms	Features
	• Reported in 69% of adults and 43% of children with TSW (3)
Swelling/Edema (3, 4, 9, 11)	• Reported in 43.3% of patients with TSW (4)
Nerve pain (3)	• May be described as “sparklers” or “zingers” (3)
Lymphadenopathy (3)	• Reported in 57% of adults and 54% of children with TSW (3)
Appetite changes (3)	• Not described in detail • May be caused by psychological changes
Psychological changes	Features
Intense emotional fluctuations (3), depression (3, 9) anxiety (3, 11)	• Reported in 81% of adults and 56% of children with TSW (3)
Suicidality (3)	• Reported in 47% of adults presenting with TSW (3)
Insomnia, sleep disturbances, or altered circadian rhythm (3, 11)	• Reported in 80% of adults and 62% of children with TSW (3)
Fatigue (3)	• Reported in 79% of adults and 53% of children with TSW (3)
Pediatric signs and symptoms	Features
Subjective growth delays (failure to meet milestones, decreased weight gain) (12)	• A systematic review of 27 pediatric patients with TSW reported 7 patients (26%) with subjective growth delays • May occur because of HPA suppression or avascular necrosis

In patients with TSW, correlating clinical signs and symptoms with a detailed history of TCS use is essential. Key elements for identifying TSW include the specific TCS used, their potency, dosage, frequency, and duration of application. However, it is important to note that limited health literacy can hinder patients' ability to recall these details, complicating diagnosis. A 2012 review found that up to 48% of patients lack functional health literacy (34), which can make it challenging to attain accurate histories.

### 4.2 Histological features

The histological features of TSA/TSW are non-specific, rendering diagnosis by histology alone improbable. In a former study, biopsies from 4 patients with TSW demonstrated spongiosis and parakeratosis; however, these histological slides were excluded from the study (21). Nevertheless, research on TSA/TSW has identified some common histological findings between the two subtypes of TSW. Both subtypes have demonstrated dilated vessels in the dermis and collagen degeneration (4).

Additionally, histological analysis of the erythematoedematous subtype revealed a thinned epidermis, spongiosis, a thin or absent granular layer, sparse perivascular infiltrate, and prominent sebaceous glands surrounded by inflammatory cells. On the other hand, the papulopustular subtype, akin to rosacea, displayed a perifollicular or granulomatous infiltrate containing neutrophils and lymphocytes (4).

## 5 Differentiating TSW/TSA from other related conditions

### 5.1 Topical steroid damaged/dependent face

In recent years, instances of improper TCS use on the face have risen in India, prompting dermatologist Koushik Lahiri to introduce the term “topical steroid damaged/dependent face (TSDF)” in March 2008. Lahiri defined TSDF as “the semi-permanent or permanent damage to the skin of the face precipitated by the irrational, indiscriminate, unsupervised, or prolonged use of TCS resulting in a plethora of cutaneous signs and symptoms and psychological dependence on the drug” (35). Although TSDF shares characteristics with TSA, such as dependency on TCS and exacerbation of cutaneous symptoms upon withdrawal (22), TSDF is specifically localized to the face, whereas TSA encompasses a broader dependency affecting various body regions.

### 5.2 Tachyphylaxis

Additionally, the terms tachyphylaxis and steroid phobia are often incorrectly associated with TSA/TSW. Tachyphylaxis is an acute condition characterized by reduced medication efficacy after successive dosing. It can be distinguished from TSA/TSW, as tachyphylaxis occurs before TCS withdrawal, while TSA/TSW exhibit cutaneous eruptions after withdrawal (9). Moreover, steroid phobia, defined as the fear or reluctance to use TCS, is associated with TSA/TSW. Awareness of TSA/TSW may exacerbate steroid phobia. Conversely, steroid phobia may lead to improper TCS usage that could perpetuate TSA/TSW. It is important to note that TSA is often attributed to TCS misuse or overuse, while steroid phobia results in TCS underuse (2).

### 5.3 Psoriasis

Psoriasis flares can be triggered by the abrupt cessation of systemic or topical corticosteroids (36). Withdrawal of these medications may lead to rebound psoriasis, often presenting with symptoms that are more severe than the initial presentation (37). These drug-associated flares of psoriasis should not be misinterpreted as TSW. Drug- induced psoriasis is similar to conventional psoriasis, but is associated with an eosinophilic infiltrate in the dermis and a lichenoid pattern on histology (36, 38).

## 6 Phases of TCS withdrawal

**Figure 4:**
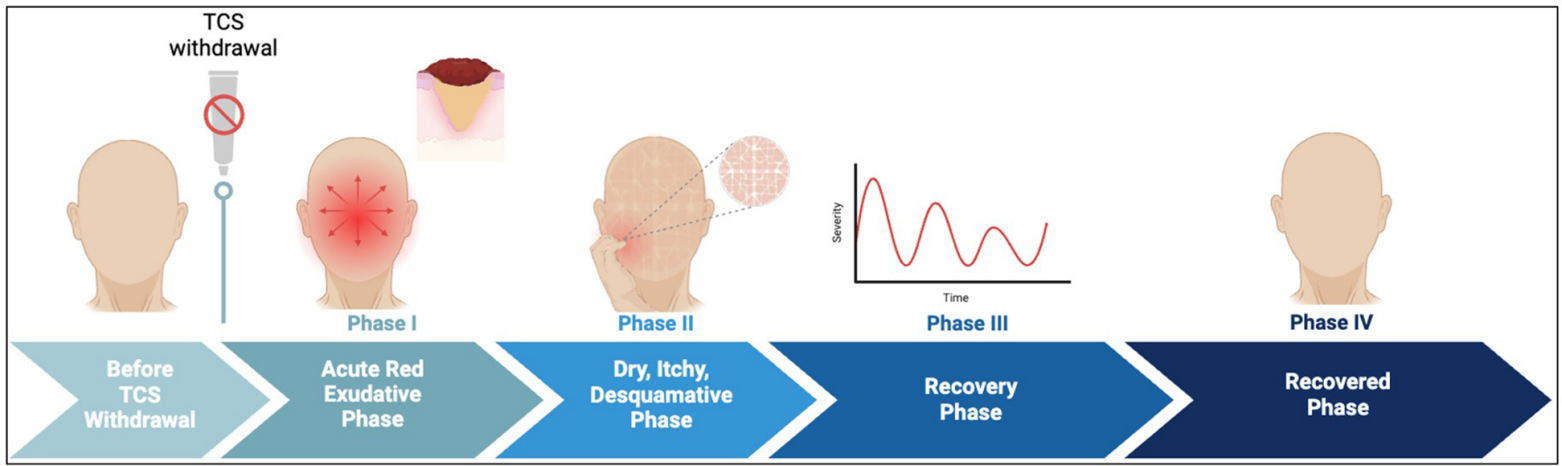
Phases of TCS withdrawal. Before TCS withdrawal patients’ skin appears normal. Phase I is characterized by acute red skin which spreads gradually. It lasts for a few days to weeks. Phase II is marked by dry, itchy, thickened skin. Some patients show signs of depression due to lack of treatment and continuous worsening of skin lesion. Phase III is the recovery phase where patients show gradual improvement of skin lesion with occasional aggravated skin flares. Phase IV is the recovered phase where patients skin normalizes and regains its original appearance. This phase may take weeks to years.

Before discontinuing TCS, patients' skin may appear normal or well-controlled by TCS, although some begin to experience symptoms such as increased pruritis or diminished TCS efficacy. In certain cases, prurigo-like eruptions or intractable nodules with severe itching emerge, often signaling addiction (9). As symptoms worsen, patients may choose to discontinue TCS for various personal reasons, such as steroid phobia, or diminished benefits despite prolonged or increased usage (2, 12, 39). TCS discontinuation is particularly relevant for patients with recalcitrant eczema, where withdrawal is a critical aspect of management. Four phases of TCS withdrawal have been proposed from previous studies (10). The sequence of events in each phase is described and illustrated in Table 2 and Figure 4 respectively.

**Table 2:** Phases of TSW.

Phases	Features
Phase I: Acute Red Exudative Phase (4, 11)	• Begins a few days after TCS cessation, lasts days to weeks (4, 11) • Rebound eruption extending to previously untreated areas, sparing palms and soles (4, 11) • Erythema develops from areas of intractable eczema and spreads gradually (4, 11) • Thickened eczematous areas may flatten, obscuring the borders of erythema (4, 11)
Phase II: Dry, Itchy, Desquamative Phase (Acute Phase) (11)	• Skin is dry, itchy, and thickened or desquamative (11) • Patients may experience depression and pessimism due to symptoms or lack of effective treatments offered by physicians (4)
Phase III: Recovery Phase (11)	• Gradual skin improvement (11) • Sensitivity to minor stimuli that gradually decreases (11) • Intermittent periods of aggravation and flares (11)
Phase IV: Recovered Phase (11)	• May take weeks to years- over time, the skin normalizes, and the increased hypersensitivity following withdrawal decreases (4, 11) • Complete withdrawal of the offending TCS leads to the skin regaining its original appearance or returning to the pre-TSA/TSW condition (e.g., AD) (4, 11) • Some patients achieve completely healthy skin if the eruption was caused by TSA rather than the underlying condition (4)

## 7 Diagnosing TSA/TSW

The non-specific features of TSA/TSW can propagate misdiagnosis and mistreatment. Several conditions can be considered in the case of TSA/TSW, such as rosacea, cutaneous T-cell lymphoma, and psoriasis (13). However, the 3 main differential diagnoses include eczematous/AD flares, allergic contact dermatitis (ACD), and infection (2).

Differentiating TSW from a flare-up of the underlying disease may be challenging but is of utmost importance. Misdiagnosing a withdrawal episode as an AD flare can lead to inappropriate treatment, often escalating TCS use. Conversely, AD flares can be misinterpreted as TSW. Both scenarios result in insufficient treatment, worsened patient outcomes, and unnecessary morbidity (6). No consensus criteria for distinguishing TSW from eczematous flares have been established. Nonetheless, previous studies suggest 3 essential diagnostic criteria for favoring a diagnosis of TSW over worsening AD:

1. Burning (4, 13) or itch (13, 21) as the prominent symptom
2. Erythema- confluent redness within days to weeks of TCS withdrawal (4, 13, 21)
3. History of prolonged, frequent TCS use on the affected region (typically the face or genitals) (4, 13, 21)

Additionally, to rule out ACD to an active steroid molecule or its vehicular excipients, patch testing may be indicated (4, 12). Localization of a rash can help identify the offending allergen or point clinicians toward suspecting ACD, such as supraumbilical dermatitis from ACD to nickel in a belt buckle (1). Despite challenges, like lack of clear skin for testing in atopic individuals (13), and potential false positives due to vasodilation (10), patch testing can determine reactions to the active steroid, its vehicle, or other environmental allergens. Given a high clinical suspicion of TSW, patch testing should be performed with the awareness of potential delayed positive reactions (13), as ACD is a type IV delayed hypersensitivity reaction. When indicated, patch testing should be administered, as mistaking ACD for TSW could prevent patients from receiving necessary anti-inflammatory therapy (4).

## 8 Treatment

**Figure 5:**
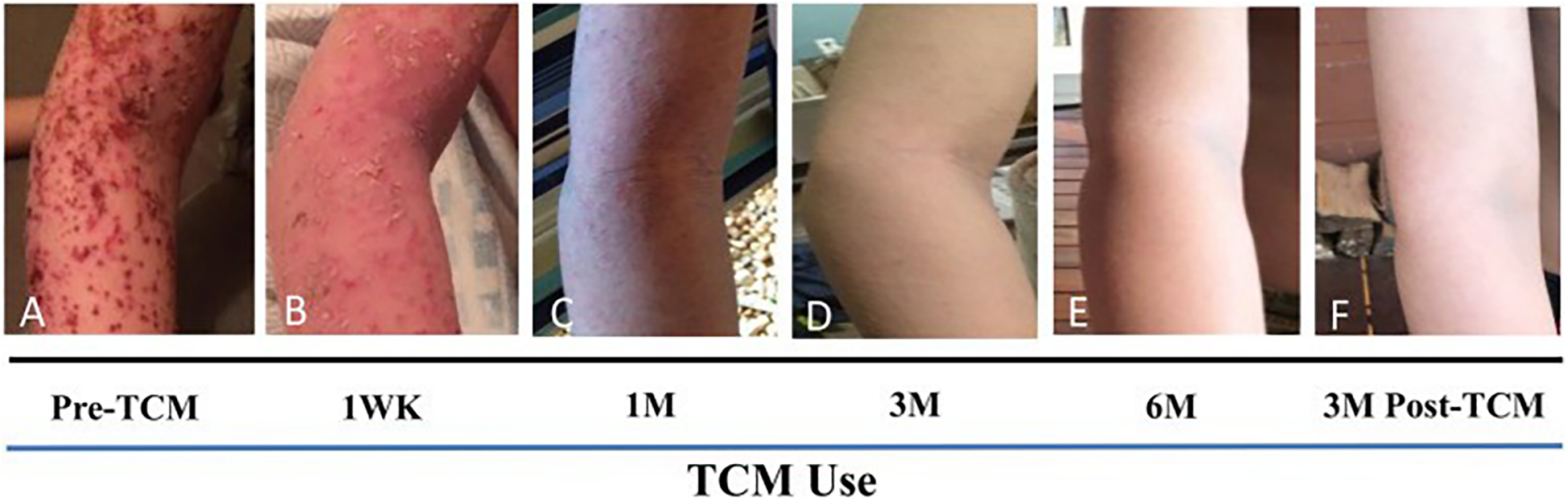
TCM therapy showed marked improved skin lesion. **(A)** 12 days after the last course of prednisone, with MSSA *Staphylococcus aureus* infection and poor response to steroid injection. **(B)** Improvement of skin lesions by 1 Week of TCM use including internal tea, external herbal bath and creams. **(C)** 1 month of TCM use, **(D)** 3 months of TCM use, **(E)** By 6 months, skin remained to be well controlled. No steroids or antibiotics were used during the course of TCM therapy. **(F)** Skin reveals no apparent recurrence 3 months after TCM discontinuation. *Figure permitted to use from* (45).

Despite the lack of general guidelines for treating TSA/TSW, several treatment approaches have shown potential value for both physicians and patients. The most widely recommended intervention is TCS discontinuation, which is advised in nearly all cases of TSA/TSW. In addition, some physicians may prescribe immune-modulating agents such as oral steroids, monoclonal antibodies, non-steroidal anti-inflammatory drugs (NSAIDs), among others. In addition, use of multi-component TCM therapy, complementary integrative medicine, black tea, coal tar and tannins are widely recognized in the literature. A summary of all these immune-modulating therapies, along with other recommended treatments, is provided in Table 3 and Figure 5.

**Table 3:** Treatment of TSA/TSW.

Treatment	Details	Adverse effects
TCS discontinuation	• In almost all cases of TSW, discontinuation of TCS is recommended • It has been suggested that a negligible difference exists between gradual tapering and immediate cessation, but patients with severe TCS addiction are more likely to benefit from immediate TCS cessation (9)	• Discontinuation may initially worsen withdrawal effects
TCS discontinuation with oral steroid supplementation	• Discontinuation of TCS followed by supplementation with systemic steroids during withdrawal rebound appears to be an effective treatment • The mechanism for this effect is currently unknown, but it is thought that systemic steroids reduce inflammatory responses with fewer dermatologic effects (9)	• Weight gain, mood changes, and increased susceptibility to infections
NSAIDs	• Along with discontinuation of TCS, the most common treatments for TSW involve agents which either dampen the immune response or have anti-inflammatory effects on the skin	• In the United States, TCIs such as tacrolimus carry a Boxed warning due to a potential for increased risk of cancer (40)
Antibiotic treatment: doxycycline, tetracycline, and erythromycin	• Frequently used in patients with the papulopustular subgroup, with oral antibiotics being more common (4) • Doxycycline, tetracycline, and erythromycin are often used for this purpose—used in 45.5% of cases (4)	• Overuse carries an increased risk of iatrogenic infection—particularly with *C. difficile* • Caution in antibiotic selection is warranted during pregnancy, as some antibiotics can be teratogenic or have unwanted side-effects
Supportive therapy (antihistamines, ice/cool compresses, etc.)	• Patients with the erythematoedematous subgroup benefited from supportive therapy with antihistamines and ice/cool compresses (4)	
Dupilumab (Dupixent®)	• A monoclonal antibody which blocks interleukins 4 and 13 • One case series showed marked improvement with dupilumab compared to other standard therapies (41)	• Some patients may experience an increased risk of headaches and conjunctivitis (42) • Some studies have shown an increased risk of certain types of cancers such as cutaneous T-cell lymphoma in patients taking dupilumab for AD (43)
Complementary integrative medicine	• Triple therapy containing traditional chines medicine (TCM) formula in the form of digestion tea, bath additives and cream has shown to markedly improved skin lesion, itching, and sleep loss in patients with TSW (44, 45) (Figure 5). • Berberine, a natural alkaloid, showed to inhibit pro-inflammatory response associated with S. aureus isolated from TSW patients (46, 47). • Multi-component TCM therapy improved SCORAD, TEWL and significantly reduced abundance of S. aureus burden and increased alpha-diversity in the skin (48). • Treatments include apple cider vinegar baths, gauze wrapping, cool compresses, hot packs, and narrowband UVB light (12) • Recent preliminary studies have shown possible benefits to increasing skin and gut microbiome biodiversity by using specialized body washes or personalized probiotics (32) • Acupuncture therapy improves clinical efficacy of itch (49).	
Black tea formulation	• Some studies have shown black tea dressings to be an effective treatment option for facial dermatitis within only a few days (50).	
Coal tar	• The topical application of coal tar is one of the oldest known treatments for AD, but its mechanism of action remains elusive (51).	
Tannins	• The benefits of tannins have been well-characterized in recent years for their anti-microbial, anti-pruritic, and anti-inflammatory effects, especially when applied topically to soothe inflammatory skin conditions (52).	
Basic skin care	• Basic routine skin care is essential for the management of eczema-related conditions. One study suggests applying 250–500 g of emollients topically each week as first-line therapy to manage inflammatory skin conditions (53).	

## 9 Prevention

**Figure 6:**
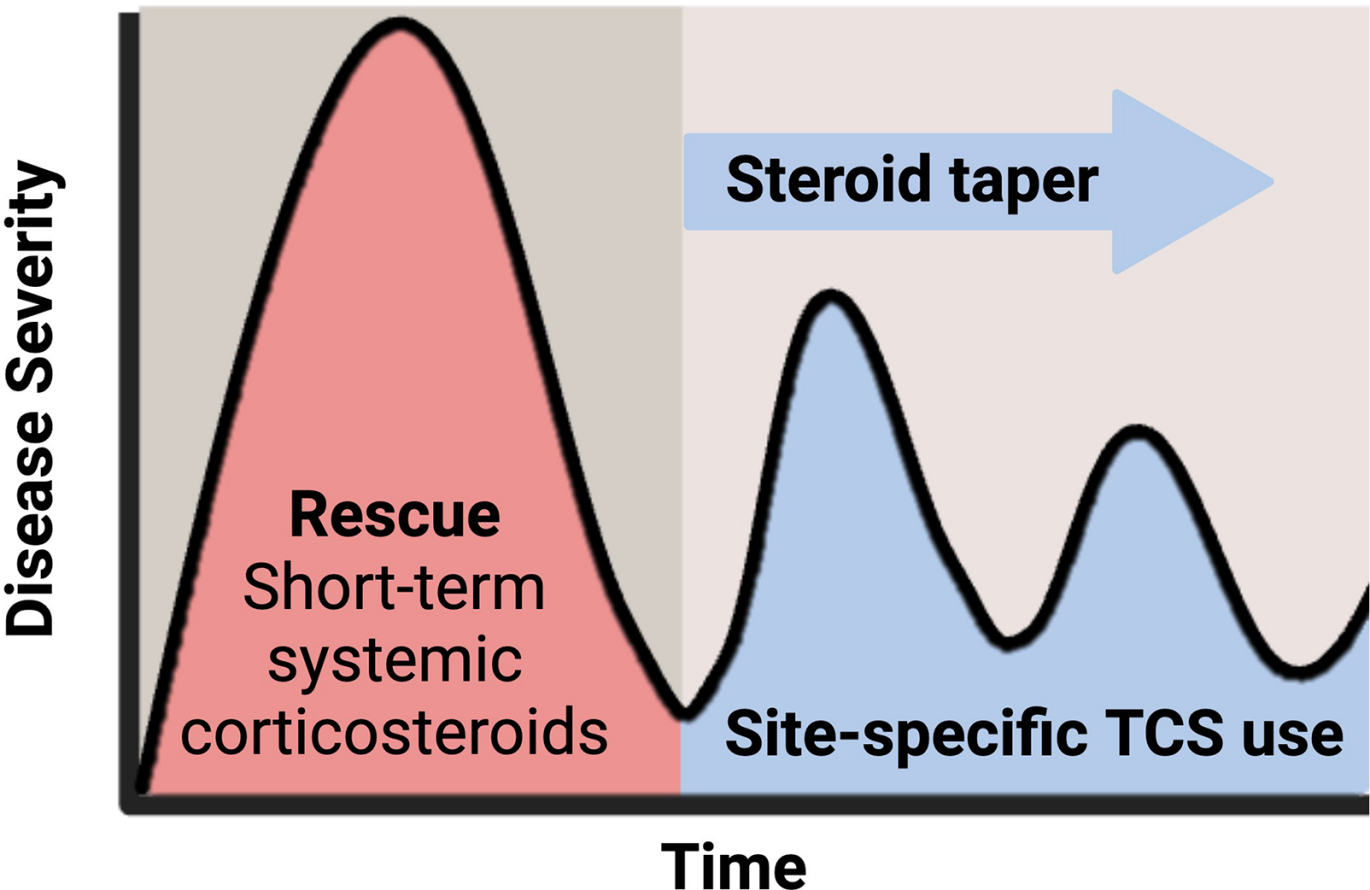
Damping moderate to severe AD with corticosteroid. Topical corticosteroid use should be limited to 2–4 weeks for appropriate response. Tapering steroid dose, followed by site-specific corticosteroids may help dampen AD severity by reducing the need for corticosteroids and limiting adverse effects like TSW.

To prevent TSA/TSW, it is recommended that continuous TCS use be limited to 2–4 weeks to prevent long-term histological effects. Periods of interruption where TCS use is discontinued may allow the skin to recover from the previous TCS cycle to permit continuation of treatment (9). Additionally, another method of preventing TSW is by dampening moderate or severe AD before disease escalation. In such cases, administering a short-term systemic corticosteroid as a rescue therapy, followed by site-specific TCS, and tapering of all steroids may be appropriate (Figure 6). In doing so, the severity of AD should dampen with time, reducing the need for corticosteroids and limiting adverse effects like TSA/TSW (6).

## 10 Prognosis and psychological impact

Active TSA/TSW symptoms can persist even for 20 months after discontinuing the offending agent (12). One survey found that 26% of patients with TSA/TSW who had stopped TCS for over 5 years still experienced symptoms. Not only can TSA/TSW be long-lasting, but it also has a sizeable psychological impact. Concern over TSW was high among adults with AD and caregivers of children with AD, with 74% of adults and 49% of caregivers reporting concern about TSW. Alarmingly, 47% of adults with symptoms consistent with TSW reported having suicidal thoughts (3). Given that TSW can persist for months to years, and is associated with high levels of concern, suicidal ideation, and significant psychological toll, frequent check-ins are crucial for evaluating and maintaining patients' emotional well-being (2). Despite increasing patient concern over TCS use and TSA/TSW, many patients find that they are met with a dismissive, ignorant or unempathetic response from their doctors. The response has resulted in patient mistrust of the medical profession, with one study reporting that 9 of 26 participants withdrew from typical dermatologic care. The resultant lack of validation for patient concerns has led some to discontinue TCS without guidance from providers and has led many to seek alternative care such as TCM (39).

## 11 Discussion and conclusion

Estimates of the prevalence of TSA/TSW vary widely, with studies suggesting that 12%–79% of patients with atopic dermatitis (AD) may experience TSW (3, 9). Therefore, it is necessary to calm down the underlying Th2 mediated immune response in early stages of AD development. Additionally, 47% of patients with symptoms consistent with TSW experience suicidal ideations. Despite the large potential burden, studying TSA/TSW presents several limitations, such as the unclear temporal relationship between TCS use and symptom onset (4), a predominance of female survey respondents (3), reliance on self-reported symptoms, and the absence of standardized diagnostic criteria. Given the substantial physical and psychological impact of TSA/TSW, continued research is necessary to establish consensus diagnostic criteria and effective treatment protocols. TSA/TSW share similarities with other dermatologic conditions; however, cardinal signs have been identified. Determination of consensus diagnostic criteria may thus be achieved by combination of these clinical signs with histological findings and detailed history of TCS use. Additionally, inflammatory markers involved in pathophysiology, such as NO, may enable quantitative differentiation from other conditions.

While diagnostic criteria are needed, proposals of such criteria cannot occur unless physicians, particularly dermatologists, recognize the legitimacy of TSA/TSW. Despite numerous patients sharing their experiences with chronic TCS use and withdrawal on online platforms, TSA/TSW remains a contentious topic within the dermatologic community. Therefore, emphasis should be given on educational training, patient-clinician symposiums and appropriate presentations on management of TSA/TSW are essential to ensure effective care. Well-informed providers will not only enhance patient trust and treatment adherence but also help mitigate steroid phobia and prevent therapeutic failures associated with improper TCS use. Enhanced provider education will lead to greater recognition of these conditions, drive the establishment of standardized diagnostic criteria, and ultimately improve clinical outcomes for patients.

Several corticosteroid-sparing strategies have been explored, however, there is an urgent need for continued research to develop new therapeutic regimens that effectively manage TSA/TSW with minimal side effects. Besides, use of natural active compounds based therapeutic strategies for steroid-dependent or steroid withdrawal associated with severe eczema along with early introduction of TCM in young adults with eczema is well discussed previously (14). A case series summary shows remarkable improvement of skin lesion, reduced itch sensation, and sleep improvement following initiation of TCM therapy protocol. Furthermore, patients undergoing substantial topical steroids, light therapy, and biological treatment showed no improvement in quality of life, however introduction of TCM therapy led to significantly improved skin integrity within 3 months. This evidence suggests TCM therapy has great potential in managing TSA/TSW. This should be further studies in controlled clinical studies of patients with severe refractory eczema and TSW to better achieve expected milestones. Additionally, advancing our understanding and treatment of TSA/TSW is crucial to restoring patient trust and ensuring compassionate care for those afflicted by these conditions.
